# Estrogen and Glycemic Homeostasis: The Fundamental Role of Nuclear Estrogen Receptors ESR1/ESR2 in Glucose Transporter GLUT4 Regulation

**DOI:** 10.3390/cells10010099

**Published:** 2021-01-07

**Authors:** Karen Cristina Rego Gregorio, Caroline Pancera Laurindo, Ubiratan Fabres Machado

**Affiliations:** Department of Physiology and Biophysics, Institute of Biomedical Sciences, University of São Paulo, Av. Prof. Lineu Prestes 1524, São Paulo (SP) 05508-900, Brazil; karengregoriorego@gmail.com (K.C.R.G.); panceracaroline@gmail.com (C.P.L.)

**Keywords:** estradiol, phytoestrogens, ESR1, ESR2, GLUT4, glycemic homeostasis, diabetes mellitus

## Abstract

Impaired circulating estrogen levels have been related to impaired glycemic homeostasis and diabetes mellitus (DM), both in females and males. However, for the last twenty years, the relationship between estrogen, glycemic homeostasis and the mechanisms involved has remained unclear. The characterization of estrogen receptors 1 and 2 (ESR1 and ESR2) and of insulin-sensitive glucose transporter type 4 (GLUT4) finally offered a great opportunity to shed some light on estrogen regulation of glycemic homeostasis. In this manuscript, we review the relationship between estrogen and DM, focusing on glycemic homeostasis, estrogen, ESR1/ESR2 and GLUT4. We review glycemic homeostasis and GLUT4 expression (muscle and adipose tissues) in *Esr1*^−/−^ and *Esr2*^−/−^ transgenic mice. We specifically address estradiol-induced and ESR1/ESR2-mediated regulation of the *solute carrier family 2 member* 4 (*Slc2a4*) gene, examining ESR1/ESR2-mediated genomic mechanisms that regulate *Slc2a4* transcription, especially those occurring in cooperation with other transcription factors. In addition, we address the estradiol-induced translocation of ESR1 and GLUT4 to the plasma membrane. Studies make it clear that ESR1-mediated effects are beneficial, whereas ESR2-mediated effects are detrimental to glycemic homeostasis. Thus, imbalance of the ESR1/ESR2 ratio may have important consequences in metabolism, highlighting that ESR2 hyperactivity assumes a diabetogenic role.

## 1. Introduction

In the early times of endocrinology, the hypophysis-related modulation of glycemic homeostasis started to be investigated by the 1947 Nobel Prize recipient in Physiology or Medicine, Bernard Houssay. At that time, several hormones related to hypophyseal axes had long been described as capable of modulating blood glucose, including estrogens. Indeed, estrogen participation in diabetes mellitus (DM) had already been investigated in humans and in experimental models [1]. However, despite reports that alterations in plasma estrogen concentration could correlate with alterations in blood glucose levels, a direct relationship between these variables was not easy to be established, since the changes in estrogen concentration could imply changes in several other hormonal systems, which eventually might modulate the blood glucose control. Regarding that, the knowledge of the estrogen mechanism of action and of molecular mechanisms involved in glycemic homeostasis should lead us to ultimately demonstrate that estrogen directly interferes in glycemic control.

DM can be classified into type 1 DM (T1D) and type 2 DM (T2D); the former primarily results from a lack of insulin secretion, whereas the later results from insulin resistance, which can lead to impaired insulin secretion [2]. In both T1D and T2D, insulin resistance compromises glucose utilization by muscle and adipose tissues, and that can either worsen the effects of insulin deficiency or reinforce insulin resistance, exacerbating hyperglycemia. Glucose uptake in muscle and adipose tissues depends on the insulin-responsive glucose transporter isoform, the solute carrier family 2 facilitated glucose transporter member 4 (GLUT4), codified by the *solute carrier family 2 member 4* (*SLC2A4*) gene, which thus plays a fundamental role in plasma glucose clearance [3]. In plasma glucose clearance, skeletal muscle seems to have a preponderant effect because of its larger tissue mass; however, in obese subjects, the role of adipose tissue becomes more relevant.

Estrogen receptor 1 (ESR1) and estrogen receptor 2 (ESR2) (formerly ERα and ERβ, respectively), as transcription factors, could modulate the expression of the *SLC2A4* gene, altering tissue GLUT4 content, and eventually modulate glycemic control. In recent years, we have investigated the estrogen-induced ESR-mediated regulation of *SLC2A4*/GLUT4 expression, expecting to demonstrate a direct effect of estrogen upon glycemic homeostasis, which could finally be helpful to ameliorate the diabetes condition.

In this review we focus on the estrogen-induced and ESR-mediated regulations of *SLC2A4*/GLUT4 expression and discuss the molecular mechanisms involved. In addition, we also discuss the potential implications of ESR1/2-mediated effects upon glycemic homeostasis and DM.

## 2. Early History of DM, Estrogen, and Their Relationship

The earliest report of a diabetes-like disease was found in an Egyptian medical papyrus, referred to as the Ebers Papyrus (supposedly prepared circa 1550 BC). In the 2nd century AC, Aretaeus of Cappadocia described the disease in detail and coined the term diabetes [4]. In the 19th century, Joseph Von Mering and Oscar Minkowski related DM to a deficient pancreatic humoral production [4], and in 1910, Jean Meyer coined the term insulin for this humor. Finally, Frederick Banting, John MacLeod, Charles Best and J.B. Collip (1921/1922) succeeded in preparing insulin capable of efficiently treating a young boy with diabetes [4].

In 1920, hormones from the ovaries were reported to produce “oestrus” (estrus), and the term oestrogen (estrogen) was coined. The first estrogen hormone was isolated in 1929, and after that, investigations of biological effects of estrogens improved in women and, more recently, in men. The mechanism of action of estrogens started to be investigated by Jensen in the 1960s [5,6], and the observation that estrogen could bind in macromolecules of target tissues led investigators to call these molecules estrogen receptors [7,8]. In the 1970s, estrogen-induced transcriptional activity was reported [9], and nuclear estrogen receptors were increasingly characterized. At present, two ESR subtypes (ESR1 and ESR2) and several isoforms have been described (for a review, see [10]).

In 1928, the improvement of glycemic control through injections of estrogenic substances (estrin) in women with DM was reported [11]. After that, the improvement of glycemic control and the extension of life span was observed in pancreatectomized diabetic dogs [1] and monkeys [12] treated with estrogen. Additionally, the estrogen-induced improvement of glycemic control was reported in women who developed diabetes related to menopause or ovariectomy [13]. Further investigations revealed a high incidence of both DM in women with gonadal dysgenesis [14] and glucose intolerance in children with Turner syndrome [15].

All in all, these data suggest that estrogen would be capable of exerting a beneficial effect on glycemic control, regardless of pancreatic insulin secretion; however, the estrogen-induced modulation of other hormonal systems (especially those related to the hypophysis) has also been considered until recently, compromising the statement that estrogen plays a direct effect on glycemic regulation.

## 3. The State of the Art in the Estrogen Regulation of Glycemic Homeostasis

### 3.1. Estrogen and Glycemic Homeostasis in Females

It is well demonstrated that women affected by Turner syndrome are at a higher risk for DM. In such condition, the development of insulin resistance is a feature; however, some studies have suggested that haploinsufficiency of X-chromosome gene(s) can also impair insulin secretion. In addition, because of hypoestrogenism, compensatory hypergonadotropism should not be excluded in the etiopathogenesis of DM in Turner syndrome (for a review, see [16,17]). On the other hand, estrogen replacement therapy is reported to improve glycemic control in postmenopausal or hysterectomized women [18]. Additionally, in spontaneously postmenopausal women, estrogen replacement improves glycemic control in T2D and decreases the risk of new-onset T2D (for a review, see [19]).

Interestingly, insulin resistance could also be related to hyperestrogenism as in women with polycystic ovary syndrome (PCO) [20,21,22]; however, in this condition, the involvement of hyperandrogenism should not be discarded (for a review, see [23]). Similarly, during pregnancy, hyperestrogenism could be related to the development of insulin resistance, both inducing gestational DM and worsening glycemic control in pregnant women with previous DM [24,25]. Nevertheless, once more the participation of other gestational diabetogenic hormones should not be discarded (for a review, see [26]). Moreover, changes in metabolic control in women with DM have been described throughout the menstrual cycle [27]. Finally, in women without DM, steroidal contraceptive treatment has been associated with increased insulin resistance, with either contraceptives containing estrogen alone or combined contraceptives (for a review, see [28]).

Altogether, these data suggest that, in women, estrogen intake can have opposite effects according to the previous circulating estrogenic (low or high) levels, highlighting the complexity of these effects.

### 3.2. Estrogen and Glycemic Homeostasis in Males

Estrogens have been associated with the female reproduction system, but studies over the last two decades have established that estrogens and their receptors ESR1 and ESR2 also regulate male reproductive and nonreproductive biological systems (for a review, see [29]).

Regarding glycemic homeostasis, decreased estrogen action secondary to a mutation in the estrogen-receptor gene in a man was reported to cause insulin resistance [30]. After that, congenital estrogen deficiency in males has been related to insulin resistance, hyperinsulinemia and even hyperglycemia in subjects with mutations in genes of estrogen receptors or aromatase (for a review, see [31,32]).

Additionally, estrogen plus antiandrogen therapy in male-to-female transsexuals has been reported to increase subcutaneous and visceral fat and to decrease insulin sensitivity [33]. Indeed, increased visceral adipose tissue with lower endogenous estrogen levels was proposed to be related to higher insulin resistance in males as compared with females [34].

Thus, as commented on for females, also in males the role of estrogen hormone in glycemic homeostasis seems to be complex, suggesting the participation of mechanisms still unknown, which might explain apparent controversial modulations.

### 3.3. Estrogen Receptors (ESRs)

Estrogen action is mainly mediated by two estrogen receptors, ESR1 (formerly ERα) and ESR2 (formerly ERβ), codified by different genes (*ESR1* and *ESR2*), which belong to the nuclear receptor family of transcription factors. ESRs contain four domains: the central DNA-binding domain (DBD), the COOH-terminal ligand-binding domain (LBD) and two activation function (AF) domains: the constitutively active AF-1 (at the NH2-terminus) and the ligand-dependent AF-2 (at the COOH-terminus) [10]. Although ESR1 and ESR2 seem to have similar affinity to estradiol (E2) and bind the same DNA response elements, they have low similarity in the AFs domains, which can change their capacity to recruit a range of co-regulatory protein complexes, and that may greatly change their final biological effect [10]. In addition to the composition of co-regulatory proteins, the proportion of ESR1/ESR2 expressed in each cell is a fundamental player in the final biological effect of estrogens. In addition, splice variants have been described for both ESR1 and ESR2; however, it is still obscure when and how the variants are expressed and are functional in each cell type (for a review, see [10,35]).

ESRs can mediate estrogen effects by binding into an estrogen response element (ERE) at the promoter region of the target genes (genomic effect) [36]. The molecular dynamics of estrogen receptor DNA-binding has been described to occur both as a dimer in its complete ERE binding site and as a monomer in a ERE half-site (for a review, see [37]). ESR1 and ESR2 have been described to bind in the complete palindromic ERE consensus sequence AGGTCANNNTGACCT, in the imperfect ERE sequences or even in the preserved or not ERE half-sites [35,37,38,39,40]. Furthermore, since the C-terminal zinc binding domain is much more flexible in monomeric binding than in dimeric binding, that facilitates secondary protein interactions and favors estrogen-induced effects involving ESR monomers together with other transcriptional factors, a genomic mechanism known as ERE-dependent transactivation (for a review, see [35]).

Estrogen effects can also be determined by mechanisms not involving direct binding of the ESRs into DNA of target genes but involving ESRs at the plasma membrane site (non-genomic effects). ESRs are predominantly detected in the nucleus, but they are constantly shuttling in and out the nucleus [41]. The plasma membrane ESRs localization seems to involve posttranslational modification (such as phosphorylation) of the receptors, followed by the assembly of a protein complex with some membrane-associated proteins [42,43,44]. In this process, proteins from the proto-oncogene tyrosine-protein kinase Src family (SRC) are involved, although ESR and SRC interaction and activation are still unclear. Dephosphorylation of the SRC C-terminus seems to determine interaction with ESR, causing its phosphorylation and translocation; however, activation (dephosphorylation) of the SRC has been ascribed to ESR1 activation, thus creating an intricate circle of activations [44,45,46]. Importantly, the ESR1 in the plasma membrane and/or in its surroundings is known to interact with phosphatidylinositol 3-kinase (PI3K) increasing RAC-serine/threonine-protein kinase (AKT) activity [47], a fundamental pathway related to insulin action.

More recently, a G protein-coupled estrogen receptor (GPER1) was described and characterized as capable of displaying non-genomic actions (for a review, see [48]). However, whether the GPER1 truly has some function in vivo has been recently discussed [49]. Furthermore, as the focus of this review is on nuclear receptors, GPER1-mediated effects of estrogen will not be discussed.

### 3.4. Glucose Transporter GLUT4

The glucose transporter protein GLUT4 was cloned in the late 1980s and belongs to a family of proteins responsible for glucose facilitative diffusion across the plasma membrane (for a review, see [50]). It is considered as an insulin sensitive glucose transporter because it mainly expresses in the classic insulin sensitive tissues such as skeletal and adipose tissues, where it is responsible for the insulin-induced glucose uptake. In myocytes and adipocytes, the binding of insulin in its receptor at the plasma membrane (PM) triggers the activation of an exquisite intracellular sorting of signals which, eventually, culminates with GLUT4 storage vesicle (GSV) translocation to the inner face of the PM. After docking and fusing events, the density of GLUT4 in the PM increases, enhancing the glucose influx. Since intracellular consumption of glucose is high in these cells, maintaining low intracellular concentrations, the diffusion gradient continuously favors the influx of the substrate. Disruption of the insulin stimulus leads to the internalization of GLUT4, restoring the glucose uptake to basal levels (for a review, see [51]). The GLUT4-mediated increase of glucose uptake in muscle and adipose tissues is a fundamental mechanism involved in blood glucose clearance, especially in the postprandial state.

Since the establishment that GLUT4 plays a fundamental role in glycemic control, we and other groups have conducted investigations on the regulation of *SLC2A4* gene expression, which codifies the GLUT4 protein [52,53,54]. At present, several transcriptional factors are clearly reported as related to the expression of *SLC2A4*/*Slc2a4* (human/murine genes, respectively), most of them acting as enhancers and a few as repressors (for a review, see [52,53]).

Interestingly, some transcriptional factors involved in *Slc2a4* expression have been related to ESR-mediated effects. However, so far, no sequence of the *Slc2a4* promoter has been confirmed as a binding site for ESR, although its promoter segment depicts some domains similar to those of the consensus binding-site (ERE). We have investigated ESR-mediated regulation of *Slc2a4*/GLUT4 expression, to be discussed in detail next.

## 4. *SLC2A4*/GLUT4 Expression and Glycemic Homeostasis

Impairment of insulin signaling transduction is a feature in insulin resistance (IR), and it can compromise PM GLUT4 translocation. This occurs in acute situations, in which the total cellular GLUT4 content is preserved. However, in a well-established chronic insulin resistant condition, reduction of GLUT4 expression is currently observed, and that certainly contributes to lower GLUT4 at the PM in response to insulin. Even considering an unaltered translocation of the GSVs, once the GSV content of GLUT4 is reduced, the final amount of GLUT4 at the PM will be reduced [55]. This fact highlights the great relevance of the repression of *Slc2a4* gene expression and eventual reduction of GLUT4 protein in the chronic IR condition related to DM. Indeed, it reinforces the importance of investigating the regulation of *Slc2a4* gene expression.

Additionally, the role of *Slc2a4*/GLUT4 expression in IR has been reinforced by studies with transgenic mice. In summary, *Slc2a4* knockout induces IR, whereas overexpression of *Slc2a4* improves glycemic control even in diabetic mice [56,57], and these regulations are directly associated to the amount of GLUT4 at the PM, independently of the alterations in the insulin signaling. Furthermore, we and other researchers have extensively reported in the literature that conditions coursing with decreased expression *Slc2a4* are accompanied by insulin resistance, whereas treatments that increase *Slc2a4* expression are accompanied by the improvement of glycemic control.

More recently, the epigenetic mechanisms involved in the regulation of *Slc2a4*/GLUT4 expression have been investigated. Some micro-RNAs, which target *Slc2a4* mRNA [58], as well as histone pot-translational modification [59] have been proposed to participate in the GLUT4 expression in DM (for a review, see [60,61]).

In view of that, we have continued to focus our studies on the regulation of the *SLC2A4* gene, considering it a promising target for the pharmacogenomics of insulin resistance [54].

## 5. Esr1, Esr2 and Cytochrome P450 Subfamily A Member 1 (Cyp19a1) Gene Manipulation Contributions

The ESR1- and ESR2-mediated participation of estrogen in glycemic regulation was greatly elucidated by studies involving spontaneous mutations of *CYP19A1* and *ESR1* in humans or gene deletion of *Cyp19a1*, *Esr1* and *Esr2* in mice. The *Cyap19a1* gene codifies the aromatase enzyme, which metabolizes androgen to estrogen; thus, impaired aromatase activity reveals a hypoestrogenic condition, in which both ESR1- and ESR2-mediated effects are expected to be impaired. Differently, *Esr1* or *Esr2* mutation or gene deletion (*ESR1* in humans) promotes a condition known as estrogen resistance in which ESR1- or ESR2-mediated effects can be selectively impaired.

### 5.1. Esr1, Esr2 and Cyp19a1 and Glycemic Homeostasis

Impaired glycemic homeostasis has been reported in men with both estrogen resistance and deficiency due to *ESR1* and *CYP19A1* gene mutations, respectively [31,32]. The generation of *Esr1*^−/−^ and *Cyp19a1*^−/−^ mice revealed that ESR1 and aromatase deficiency leads to the development of obesity and insulin resistance [62,63]. Curiously, the selective *Cyp19a1*^−/−^ deficiency in hematopoietic cells increases whole body insulin sensitivity, which has been associated with reduced estrogen generation in muscle, but not in adipose cells [64]. In addition, in *Esr2*^−/−^ mice, glucose tolerance and insulin sensitivity were described as equal or improved as compared with wild-type mice [65]. We have further investigated this field considering the ESR-mediated regulation of *Slc2a4*/GLUT4 expression, to be discussed next.

### 5.2. Esr1, Esr2 and Cyp19a1 and GLUT4

A pioneering study regarding *Esr1*, *Esr2* and *Cyp19a1* gene knockout and GLUT4 expression was published in 2006 [66]. Immunocytochemistry analysis of skeletal muscle of male mice revealed that (1) ESR1 and ESR2 co-express in the nucleus, (2) GLUT4 expression strongly decreases in *Esr1*^−/−^ mice, (3) GLUT4 expression slightly increases in *Esr2*^−/−^ mice, (4) treatment of *Cyp19a1*^−/−^ mice with the ESR2 agonist 2,3-bis(4-hydroxyphenyl)propionitrile (DPN) strongly reduces GLUT4 expression and (5) treatment of *Cyp19a1*^−/−^ mice with the ESR1 agonist 4,4′,4”-(4-propyl-1H-pyrazole-1,3,5-triyl)trisphenol (PPT) increases expression and concentrates the GLUT4 at the PM [65]. All in all, this indicates that ESR1 enhances and ESR2 represses GLUT4 expression, with a predominant effect of ESR2 on the skeletal muscle cell. Additionally, reduction in *Slc2a4* mRNA expression was also detected in subcutaneous and visceral adipose tissues of *Esr1*^−/−^ female mice [67]. Interestingly, although global aromatase deficiency does not significantly modify muscle GLUT4 expression [66], selective aromatase deficiency in hematopoietic cells increases ESR1 and GLUT4 expression in muscle, indicating an important role of local E2 generation [64].

Later, Barros and colleagues [68] reported, in *Esr2*^−/−^ male mice, increased insulin sensitivity with fasting hypoglycemia, increased GLUT4 expression in skeletal muscle and pancreatic islet hypertrophy. Furthermore, in wild type mice, ESRs expression was clearly reported to be ESR2 < ESR1 in muscle whereas ESR1 > ESR2 in adipose tissues (for a review, see [68,69,70]).

On the whole, these studies definitely demonstrate that ESR1-mediated effects lead to improved insulin sensitivity, whereas ESR2-mediated effects are diabetogenic, highlighting that the regulation of *Slc2a4*/GLUT4 plays a key role in these effects. Furthermore, in a whole body, the final E2-induced effect must be resultant of the ESR1/ESR2 balance, accentuating that ESR1 is predominant in adipocytes whereas ESR2 is predominant in myocytes. These data collectively corroborate the complexity of the exquisite role of E2 upon glycemic homeostasis.

## 6. Estrogen-Induced Regulation of *Slc2a4*/GLUT4 Expression

Investigating the regulation of *Slc2a4*/GLUT4 expression by estrogen is always challenging, since the manipulation of estrogen concentration can induce several other metabolic-hormonal regulations, which could in fact be the true modulators of the transporter. The induction of hypoestrogenism in *Cyp19a1*^−/−^ transgenic male mice [62] and in ovariectomized rats [71] was accompanied by increased GLUT4 protein in skeletal muscle; however, insulin sensitivity decreases in *Cyp19a1*^−/−^ mice and increases in ovariectomized rats. Furthermore, during pregnancy, as circulating estrogen levels increase, insulin resistance also increases and GLUT4 expression decreases progressively [72,73]. Additionally, a transient increase in GLUT4 expression was reported in muscle of early lactating rats [72], and recently, this effect was proposed to be associated with a transient increase in ESR1 expression [74], highlighting that ESR2 is predominant in muscle. These data demonstrate the complexity of the adjustments triggered in vivo.

The estrogen-induced regulation of *Slc2a4*/GLUT4 expression started to be clarified by studies of estradiol (E2) effects on isolated target cells, mainly on adipocytes. Twenty-four-hour 10 nM E2 was reported to repress *Slc2a4*/GLUT4 in L6 myotubes [71] but to enhance *Slc2a4*/GLUT4 in 3T3-L1 adipocytes [75] and in female rat primary adipocytes [67]. These results conform with those observed in Esr1^−/−^ and Esr2^−/−^ mice: ESR2 is predominant in the myotube, whereas ESR1 is predominant in adipocytes, and ESR2 is a repressor whereas ESR1 is an enhancer of *Slc24*/GLUT4 expression.

The role of ESR1 and ESR2 in the regulation of *Slc2a4*/GLUT4 expression was thoroughly investigated in 3T3-L1 adipocytes by employing ESR1 and ESR2 agonists (PPT and DPN), as well as the ESR1 antagonist 1,3-Bis(4-hydroxyphenyl)-4-methyl-5-[4-(2-piperidinylethoxy)phenol]-1H-pyrazole dihydrochloride (MPP) and the ESR2 antagonist 4-[2-Phenyl-5,7-bis(trifluoromethyl)pyrazolo[1,5-a]pyrimidin-3-yl]phenol (PHTPP), in the presence or not of E2 [76]. The co-expression of ESR1 and ESR2 in the nucleus was detected using immunocytochemistry in E2 untreated adipocytes. It was definitely demonstrated that ESR1 enhances, whereas ESR2 represses, *Slc2a4* gene expression, regulations parallelly accompanied by GLUT4 protein expression and glucose uptake changes [76].

## 7. ESR1/ESR2-Mediated Regulation of *SLC2A4*/GLUT4

### 7.1. ESR1/ESR2 Nuclear Direct Regulation of SLC2A4 Gene

**Figure 1:**
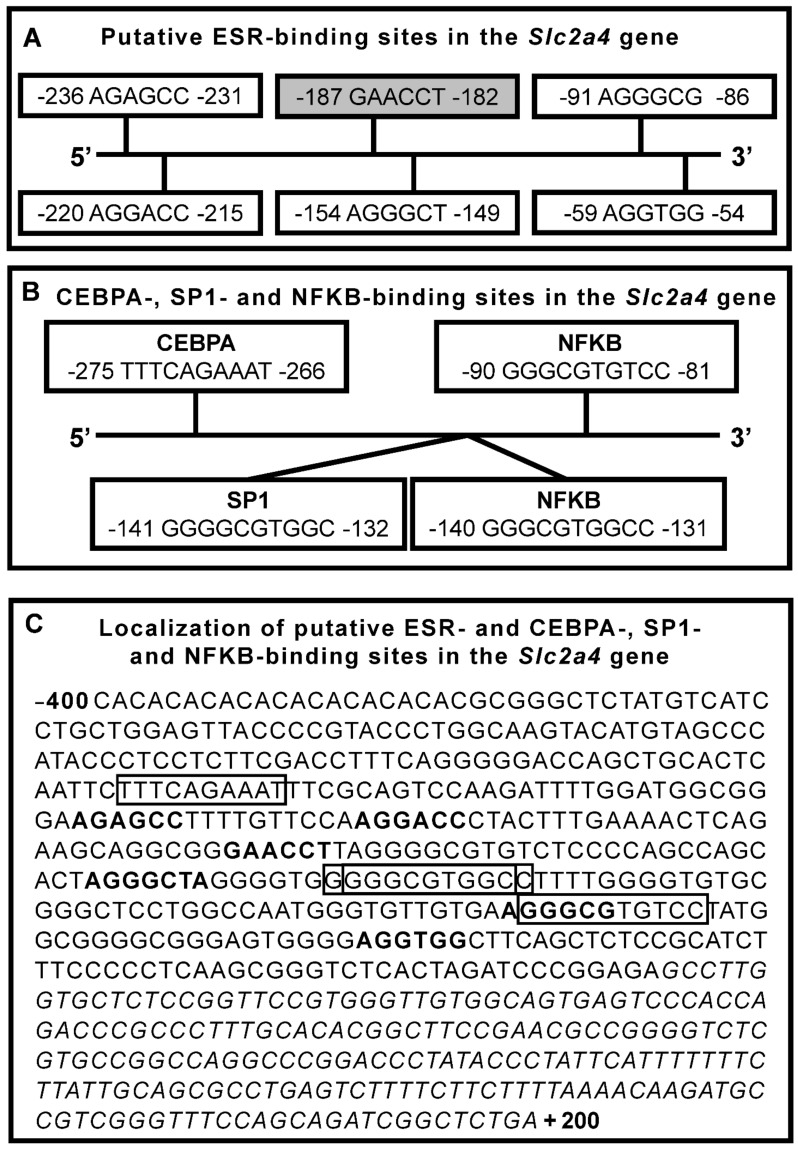
Analysis of binding sites for some transcription factors in mouse *Slc2a4* gene. (**A**) Localization of putative estrogen receptor (ESR)-binding sites in the *Slc2a4* promoter region. Based on the consensus ESR-binding site AGGTCANNNTGACCT, there are five short sequences similar to the first half-site (white boxes) and one short sequence similar to the second half-site (gray box). (**B**) Localization of confirmed functional binding sites for nuclear factor NF-kappa-B (NFKB), specific protein 1 (SP1) and CCAAT/enhancer-binding protein alpha (CEBPA) transcription factors in the *Slc2a4* promoter (there are two NFKB-binding sites). (**C**) Combined data from panels (**A**,**B**) reveal the proximity between the putative ESR-binding sites and the confirmed CEBPA-, SP1- and NFKB-binding sites; ESR-binding half-sites are in bold, and CEBPA-, SP1-, and NFKB-binding sites are inside the boxes (according to the positions shown in panels (**A**,**B**)). SP1- and NFKB-binding sites overlap, and the −140/−131 NFKB-binding site overlaps a putative half-site of ERE. The *Slc2a4* sequence is according to the mouse transcript ID: ENSMUST00000018710.12, from https://www.ensembl.org.

ERSR1 and ESR2 have been described to bind in ERE palindromic consensus sequence AGGTCANNNTGACCT, in imperfect ERE sequences or even in perfect or imperfect ERE half-sites [37,38,39,40]. However, the *Slc2a4* promoter contains neither the perfect consensus palindromic sequence nor the perfect half-sites. However, we can observe some putative ERE sequences in the *Slc2a4* promoter region (Figure 1A). The −245/−29 segment of the mouse promoter region of the *Slc2a4* gene (transcript ID: ENSMUST00000018710.12; from https://www.ensembl.org) depicts (1) five sequences similar to the first half-site of the consensus ERE (one with 50% and four with 67% of similarity), (2) one sequence similar to the second half-site of the consensus ERE (with 67% of similarity) and (3) one sequence similar to the consensus palindromic ERE (with 60% of similarity) (Figure 1A). None of these putative ESR-binding sites have been evaluated concerning their binding to ESR1/2 and transcriptional activity, thus requiring further investigation.

Additionally, *Slc2a4* gene expression can also be regulated by ESR1/ESR2 genomic effects that occur in cooperation with other transcriptional factors [35], to be discussed next as an indirect mechanism.

### 7.2. ESR1/ESR2 Nuclear Indirect Regulation of SLC2A4 Gene

Estrogen-induced effects involving ESRs monomers and other transcription factors are known as ESR-dependent transactivation [39]. Currently, these indirect effects occur through protein-protein interaction, in which the transcriptional factor, but not the ESR, binds in the DNA of the target gene (for a review, see [35]).

Additionally, interactions between an ESR monomer and a transcription factor, both bound into the DNA, have already been described [40,77]. Since in this situation the transcriptional activity cannot be triggered solely by the ESR, we will consider it as an indirect regulation, despite the binding of the ESR monomer.

#### 7.2.1. Nuclear Factor NF-Kappa-B (NFKB)

The NFKB family includes proto-oncogene c-Rel (c-REL), transcription factor p65 (RELA/p65), transcription factor RelB (RELB), nuclear factor NF-kappa-B p105 subunit (NFKB1/p105) and nuclear factor NF-kappa-B p100 subunit (NFKB2/p100) proteins, codified by *REL proto-oncogene*, *NF-kappa-B subunit* (*REL*), *RELA proto-oncogene*, *NF-kappa-B subunit* (*RELA*), *RELB proto-oncogene*, *NF-kappa-B subunit* (*RELB*), *nuclear factor kappa-B subunit 1* (NFKB1) and *nuclear factor kappa-B subunit 2* (*NFKB2*) genes, respectively. Besides, p105 and p100 may generate the respective p50 and p52 subunits. The nomenclature of proteins and genes are in accordance with UNIPROT (http://www.uniprot.org) and HGNC (http://www.genenames.org) databases, respectively. All subunits have a kappa-B domain which shares an NFKB binding site in target genes. NFKB proteins play a role as homo- or heterodimers complexes; p65/p50 heterodimer is the most abundant, especially in adipose and muscle tissues (http://www.uniprot.org).

NFKB has been extensively related to directly regulating *Slc2a4* gene expression. We have reported that increased NFKB activity participates in the repression of *Slc2a4*/GLUT4 expression induced by inflammation, oxidative stress and endoplasmic reticulum stress [78,79,80,81,82], whereas decreased NFKB activity participates in the enhancement of *Slc2a4*/GLUT4 expression induced by insulin [79,82,83]. Although the *Slc2a4* gene does not display a consensus NFKB-binding site, our group demonstrated the sequence and localization of two NFKB-binding sites in the *Slc2a4* promoter (Figure 1B), which were confirmed to bind p65 and p50 and to repress *Slc2a4* transcription, both in muscle and adipose tissues [78].

Interaction between ESR and NFKB was first reported to be an ESR-induced impairment of the c-REL and, to a lesser extent, of the p65 binding in the *interleukin 6* (*IL6*) promoter gene [84]. After that, inhibitory reciprocal interactions between ESR and NFKB have been extensively reported. The trans-repressive interaction between ESR and NFKB may involve several mechanisms such as (1) activation of the PI3K signaling pathway, leading to the accumulation of NFKB in the cytosol, (2) direct repression of NFKB binding into the DNA, (3) interaction with NFKB co-repressors and (4) competition for NFKB co-activators (for a review, see [85]). Interestingly, although rare, evidence of a synergistic positive interaction between ESR and NFKB has already been reported, which results in an increase in NFKB binding [86].

**Figure 2:**
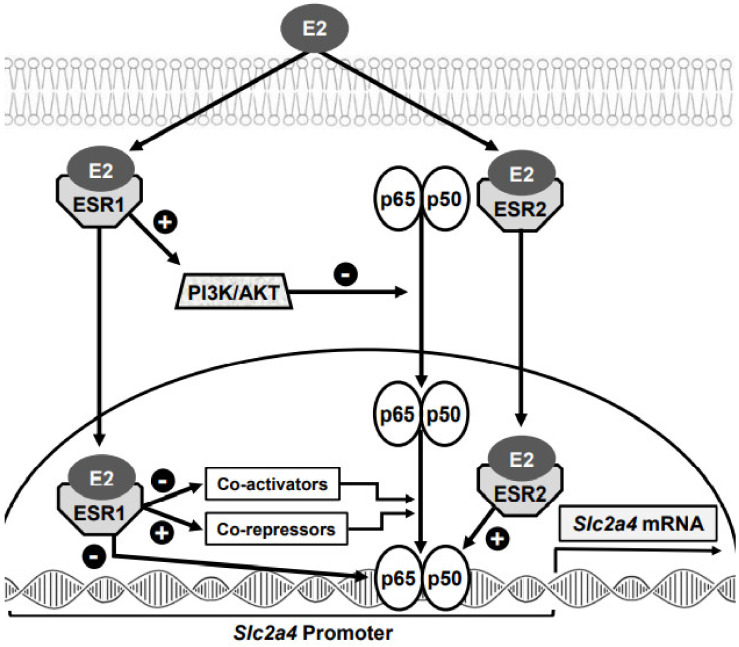
Model representing the mechanisms through which the nuclear factor NF-kappa-B (NFKB) can participate in the E2-induced and ESR1/ESR2-mediated regulation of *Slc2a4* gene transcription. E2 binds and activates ESR1 in the cytosol; thus, ESR1 activates the phosphatidylinositol 3-kinase (PI3K)/RAC-serine/threonine-protein kinase (AKT) pathway, which in turn inhibits the NFKB (p65/p50) translocation to the nucleus. In the nucleus, ESR1 can (1) directly repress the p65/p50 binding into the DNA, (2) interact with NFKB co-repressors increasing their activity and (3) compete with NFKB co-activators, reducing their activity. E2-induced activation of ESR2 in the nucleus promotes a synergistic positive interaction increasing NFKB (p65/p50) binding into the DNA. Considering that the NFKB is a repressor of *Slc2a4* transcription, the ESR1-induced reduction and the ESR2-induced increase in NFKB activity can explain, respectively, the enhancement and repression of *Slc2a4* transcription. The resultant will depend on the ESR1/ESR2 balance in the target cell.

The participation of NFKB in the E2-induced regulation of *Slc2a4* gene expression was determined by analyzing the NFKB (p65/p50) binding activity into the *Slc2a4* promoter in adipocytes treated with E2 and selective agonist/antagonist of ESR1 and ESR2 [76]. NFKB binding activity into *Slc2a4* promoter is strongly decreased by ESR1 stimulation, revealing the classic trans-repressive interaction between ESR1 and NFKB. Considering that NFKB is a repressor of the *Slc2a4* gene; consequently, the ESR1-induced enhancement of the gene expression can be explained. On the other hand, the expected ESR2 synergistic positive effect upon NFKB activity was clearly observed by the addition of E2 in ESR1 blocked cells (favoring ESR2 activation); this increased NFKB binding activity may explain the ESR2-induced repression of *Slc2a4* transcription [76]. Based on these data, and on the mechanisms of ESR/NFKB interactions described, NFKB participation in the ESR1/ESR2-induced regulation of *Slc2a4* gene expression is summarized in Figure 2.

#### 7.2.2. Specific Protein 1 (SP1)

ESR1 and ESR2 are known to interact with SP1, modulating the expression of several target genes. This involves the binding of both ESR and SP1 into their cognate DNA elements; ESR usually binds in half-site motifs (for a review, see [40,77]). However, ESR/SP1 interactions in which only SP1 binds into the DNA have also been described (for a review, see [40,77]). In addition, ESR1/SP1 interaction is known to transactivate genes, whereas ESR2/SP1 interaction is primarily associated with the repression of target genes [40,77]. Furthermore, in these regulations, E2-induced activation of ESRs promotes the translocation and accumulation of SP1 in the nucleus [87].

The most common mechanism of ESR/SP1 interaction involves the binding of both ESR and SP1 in the DNA, in specific ESR and SP1 binding motifs close to each other, separated by 3 to 68 nucleotides [40]. SP1 is a classic enhancer of *Slc2a4* transcription, and an SP1 binding site of mouse *Slc2a4* promoter is shown in Figure 1B [88]. Interestingly, the SP1 binding site is located close to several putative ESR binding half-sites: two up to 73 nucleotides upstream and two up to 72 nucleotides downstream of the SP1 binding site (Figure 1C). In addition, one first half-site of the ESR binding is separated from the SP1 binding site by only 6 nucleotides (Figure 1C). That makes the SP1/ESR cooperativity highly probable in *Slc2a4* gene expression.

**Figure 3:**
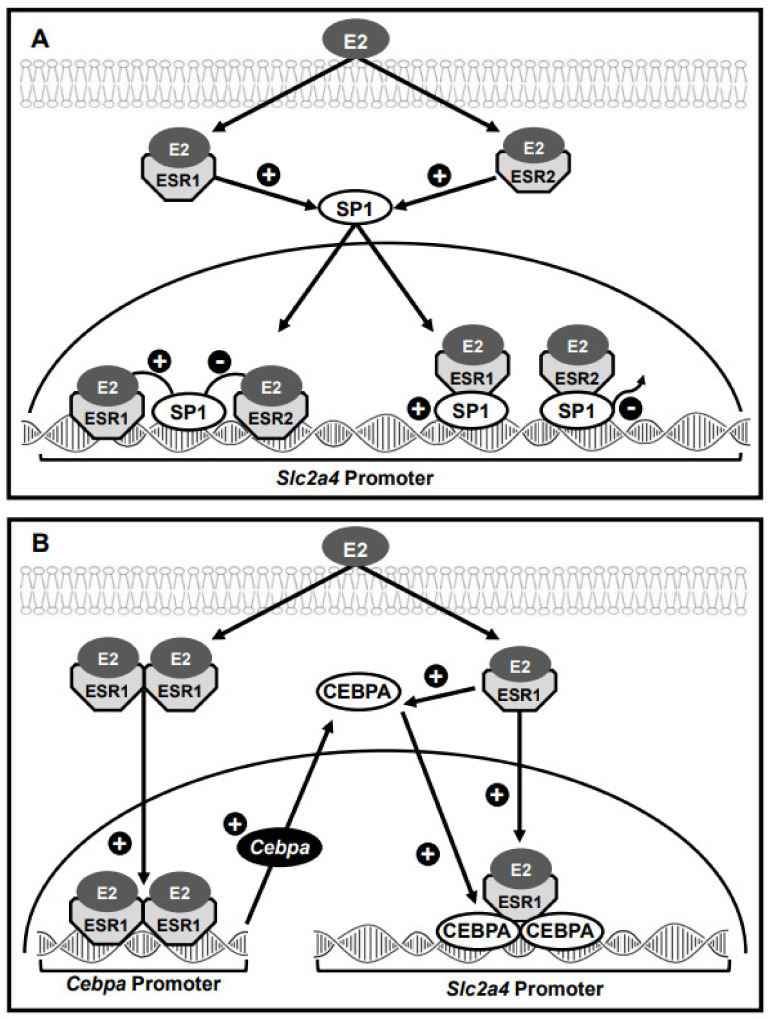
Models representing mechanisms through which the specific protein 1 (SP1) and the CCAAT/enhancer-binding protein alpha (CEBPA) can participate in the E2-induced and ESR1/ESR2-mediated regulation of *Slc2a4* gene transcription. (**A**) SP1 participation: E2-induced activation of both ESR1 and ESR2, in the cytosol, stimulates SP1 translocation to the nucleus. In the nucleus: (1) ESR1 and ESR2 bind into the DNA, close to the SP1-bound protein, and respectively stimulate and repress the SP1 enhancer activity; (2) ESR1 and ESR2 interact with SP1 protein, stimulating (ESR1) or inhibiting (ESR2) SP1 binding into the DNA. SP1 activation enhances *Slc2a4* transcription. (**B**) CEBPA participation: (1) E2 activates ESR1 dimerization, nuclear translocation and binding into the *Cebpa* gene promoter, leading to increased *Cebpa* mRNA transcription and further CEBPA protein translation; (2) E2-induced activation of ESR1 in the cytosol stimulates the nuclear translocation of CEBPA; (3) in the nucleus, ESR1 interacts with CEBPA and stimulates its binding into the *Slc2a4* promoter. CEBPA activation enhances *Slc2a4* transcription.

In isolated adipocytes, 24-h stimulus with selective ESR1 and ESR2 agonists increases the nuclear content of SP1, without changing the nuclear content of ESR1 and ESR2 [89]. ESR1 activation also increases the physical interaction between ESR1/SP1 in the nuclear protein extract (SP1 immunoprecipitation followed by immunoblotting with anti-ESR1) and eventually increases SP1 binding into the *Slc2a4* promoter [89]. This reveals the participation of SP1 in ESR1-induced activation of *Slc2a4* expression. On the other hand, the effects of ESR2 activation were not clear; however, the addition of E2 to the ESR1 agonist reversed the increased SP1 binding activity, suggesting an opposite ESR2-mediated effect. The participation of these mechanisms in the *Slc2a4* expression is summarized in Figure 3A.

#### 7.2.3. CCAAT/Enhancer-Binding Protein Alpha (CEBPA)

CCAAT/enhancer-binding proteins (CEBPs) are a family of six well characterized transcriptional factors (for a review, see [90]). The isoforms usually act as homodimers, although, because of a highly conserved bZIP domain, they can form heterodimers in all intrafamilial combinations (except with CEBPZ), always interacting with an identical binding site. CEBPA, CEBPB and CEBPD play a fundamental role in adipogenesis: CEBPB and CEBPD are expressed early in the adipogenesis process, throughout mitotic clonal expansion, whereas CEBPA is expressed late, during the adipocyte differentiation [90]. Adipocyte differentiation starts with *Slc2a4*/GLUT4 expression, for which the CEBPA plays an important role [91,92,93], and a CEBPA binding site at the mouse *Slc2a4* promoter (Figure 1B,C) was clearly reported to act as an enhancer in *Slc2a4* gene transcription [91].

ESR1/CEBPA interaction has been described to participate in the E2-induced regulation of several target genes, including genes related to metabolism such as *insulin growth factor 1* (*Igf1*) [94] and *resistin* (*Retn*) [95]. More recently, CEBPA was proposed to participate in the E2-induced modulation of adipose tissue mass in ovariectomized rats [96].

Recently, we have thoroughly investigated the participation of ESR1-mediated E2 regulation of 3T3-L1 adipocyte differentiation [67]. From days zero to eight of 3T3-L1 differentiation, E2 increased (1) adipocyte maturation (lipid content), (2) *Cebpa* mRNA expression and nuclear content of CEBPA protein, (3) *Slc2a4* mRNA expression and total cellular content of GLUT4 and (4) CEBP binding into *Slc2a4* promoter. In addition, in 8-day differentiated adipocytes, 24-h E2 treatment also increased *Cebpa* mRNA, the nuclear content of CEBPA protein and the *Slc2a4* mRNA, all effects blocked by *Esr1* gene silencing. These data definitely demonstrate ESR1 participation in *Slc2a4* expression induced by E2 and mediated by CEBPA. CEBPA participation in the ESR1-induced enhancement of *Slc2a4* gene expression is summarized in Figure 3B.

#### 7.2.4. Peroxisome Proliferator-Activated Receptor Gamma (PPARG)

Increased expression of *Pparg* and *Cebpa* is a feature of the final step of adipogenesis, the terminal adipocyte differentiation, which involves the expression of *Slc2a4*/GLUT4, a marker of adipocyte maturity [97]. PPARG is considered as a regulator of *Slc2a4* gene expression (for a review, see [98]); however, unlike CEBPA (a *Slc2a4* enhancer), PPARG in its unliganded state represses transcription of *Slc2a4*, and its endogenous natural ligand prostaglandin J2 does not alter this repressor effect [99]. Thus, the parallel increase in PPARG and GLUT4 during adipocyte differentiation seems to have no relationship. Surprisingly, the addition of PPARG synthetic ligand thiazolidinediones completely alleviated the PPARG repression of the *Slc2a4* gene, and that has led these drugs to be erroneously called PPARG agonists regarding *Slc2a4* regulation [98,99].

PPARG forms heterodimers with the retinoid acid receptor RXR-alpha (RXRA), and the PPARG/RXRA complex binds into a DNA consensus sequence presenting two direct repeats of AGGTCA, separated by a single nucleotide (AGGTCANAGGTCA) [100]. In addition, it has been proposed that PPARG and RXRA display a half-site selective binding, respectively, at the 5′ and 3′ half-sites [100]. Interestingly, the first half-site of the consensus ESR binding site (AGGTCA) matches perfectly with the two half-sites of the PPAR/RXRA binding site (AGGTCA), making the ESR/PPARG/RXRA interactions highly plausible [100].

In fact, overly complex and unclear ESR1/ESR2-mediated relationships between E2 and PPARG have been reported in several situations such as breast cancer development, osteogenesis, adipogenesis and lipogenesis [101,102,103]. In general, these interactions have been described as negative crosstalk, in which ESR1/2 could repress the PPARG expression and/or activity [65,102,103]. Indeed, it has been reported that ESR1 physically interacts with the PPARG response element repressing PPARG-mediated transcriptional activity of ESR1 in the Michigan Cancer Foundation-7 (MCF7) cells [104]. Thus, we can hypothesize that the ESR1 trans-repression of transcriptional activity of PPAR (repression of *Slc2a4* transcription) might participate in the ESR-induced regulation of *Slc2a4* expression.

The transcriptional effect of PPARG upon the *Slc2a4* gene (repressor) has been extensively investigated (for a review, see [98]); however, to our knowledge, the exact PPARG binding site into the *Slc2a4* gene is still unknown. The first mechanistic study described that PPARG/RXRA binds to the −66/+163 bp segment of the mouse *Slc2a4* gene without indicating the exact binding site [99]. The in silico analysis of this region in the mouse *Slc2a4* gene sequence that we have applied (ensembl.org; transcript ID: ENSMUST00000018710.12, Figure 1C) reveals that there is neither the PPARG/RXRA binding site consensus sequence (AGGTCANAGGTCA), nor a perfect half-site sequence (AGGTCA). However, in the −66/+163 regions, we can find two segments with 67% of similarity to the AGGTCA: (1) AGGTGG (ending at −54 bp) and (2) AGGCCC (starting at +97 bp). These domains might be related to the PPARG/RXRA binding activity to *Slc2a4* already described.

Recently, the participation of PPARG in the ESR1-mediated regulation of adipogenesis was investigated during the differentiation phase (days zero to eight) of 3T3-L1 adipocytes [67]. As expected, the *Pparg* mRNA and PPARG nuclear protein content increased during adipocyte differentiation. However, although E2 improved cellular differentiation, *Slc2a4* mRNA expression and GLUT4 expression, E2 did not alter *Pparg*/PPARG expression, indicating that PPARG participates neither in the E2-induced positive effect, nor in the cellular differentiation or *Slc2a4*/GLUT4 expression [67]. Furthermore, 24-h E2 treatment of differentiated adipocyte did not alter the *Pparg* expression either, and the adipocyte *Esr1* silencing did not modify the *Pparg* expression, regardless of the addition of E2 [67]. These data indicate that the positive ESR1-mediated E2-induced effects upon adipocyte differentiation and *Slc2a4*/GLUT4 expression do not involve the participation of PPARG.

### 7.3. ESR1 Effects Associated to Its Plasma Membrane (PM) Localization

#### 7.3.1. E2-Induced Translocation of ESR1 to the PM

The capacity of ESRs to shuttle from the nucleus to the PM and thus to activate some PM-related signals was commented on above in Section 3.3. (for a review, see [105]). In adipocytes, the E2-induced ESR shuttling from the nucleus to the PM was elegantly demonstrated using immunohistochemistry [75]: in the absence of E2, both ESR1 and ESR2 are strongly detected in the nuclear compartment; after 24-h E2-treatment, a strong nuclear exclusion of ESR1, but not of ESR2, was observed [75]. Furthermore, the ESR1 translocation to the PM was perfectly mimicked by the ESR1 agonist PPT in the absence of E2, and the E2-induced ESR1 translocation was completely abolished by the ESR1 antagonist MPP. Finally, the SRC inhibitor 4-amino-5-(4-chorophenyl)-7-(*t*-butyl)pyrazolo[3,4-d]pyrimidine (PP2) completely blocked the E2-induced ESR1 translocation [75].

Notably, the PM translocation of ESR1 induced by both E2 and PPT (alone) promotes a potent activation of AKT (phosphorylation), in the same magnitude of that observed with insulin, the classic AKT activator [72]. Furthermore, E2-induced AKT phosphorylation was completely abolished by PP2, indicating that E2-induced activation of AKT is related to the PM ESR1 [75]. Importantly, this PI3K/AKT activation (induced by the PM ESR1) may be related to the activation (including translocation to the nucleus) of the transcription factors described above (Section 7.2), thus (indirectly) participating in *Slc2a4* gene expression.

In summary, in adipocytes, E2 induces (in an SRC-mediated mechanism) the ESR1 translocation from the nucleus to the PM, with no changes in the ESR2 subcellular distribution. In addition, ESR1 translocation can activate the PI3K/AKT pathway.

#### 7.3.2. E2-Induced GLUT4 Translocation to the PM

The E2-induced improvement of insulin-induced glucose uptake in adipocytes has been reported, albeit in a controversial dose-dependent pattern [106,107,108]. These studies revealed that E2 could enhance the PI3K/AKT activity and the insulin receptor substrate 1 (IRS1) activity, upstream of the PI3K/AKT activation [107,108]. Among these studies, only one tried to correlate the E2-induced activation of IRS1/PI3K/AKT with the subcellular ESR distribution, and unexpectedly, the activation of the pathway was observed concomitantly with the ESR1 nuclear restraint [108].

Considering that (1) the PI3K/AKT activation starts at the PM region; (2) E2 induces the ESR1 translocation to the PM and 3) the PI3K/AKT pathway is a classic pathway involved in insulin-induced PM GLUT4 translocation, it is reasonable to expect that E2 could stimulate PM GLUT4 translocation.

The first indication that ESR1 and ESR2 are differently involved in PM GLUT4 translocation, as they are in *Slc2a4* expression, was detected in transgenic mice. In skeletal muscle of *Cyp19a1*^−/−^ (knockout of aromatase) mice, ESR1 agonist PPT, but not ESR2 agonist DPN, stimulates GLUT4 translocation [66]. Other changes in PM GLUT4 content were described in muscles of transgenic mice, but always together with parallel changes in the total GLUT4 content [66,68]. Since variations in the total cellular GLUT4 expression reflect in parallel variations in the GLUT4 PM content, it is difficult to ascribe those variations to a specific ESR-mediated effect upon GLUT4 translocation.

**Figure 4:**
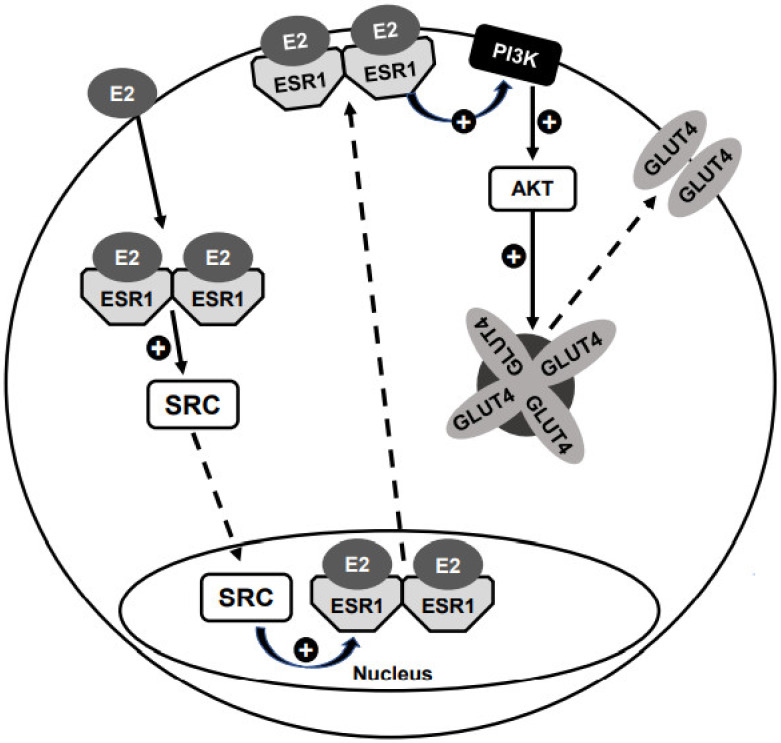
Model of E2-induced GLUT4 translocation to the plasma membrane (PM). E2-induced activation of ESR1 in the cytosol leads to the activation of proteins from the proto-oncogene tyrosine-protein kinase Src family (SRC), which in turn translocate to the nucleus. In the nucleus, SRC proteins interact with ESR1, promoting its nuclear exclusion and translocation to the PM. At the PM region, ESR1 interacts with PI3K and activates the PI3K/AKT pathway. Activation of AKT is a key step to promote the mobilization of GLUT4 storage vesicles to the PM, followed by docking and fusing the GLUT4 protein into the PM. The result is an E2-induced/ESR1-mediated increase in the cellular glucose uptake. Black arrows indicate activation and dashed arrows indicate translocation.

E2-induced PM GLUT4 translocation was finally demonstrated by studies in isolated adipocytes. Exquisite immunocytochemical images were obtained by Campello and colleagues [75] revealing that 24-h E2 treatment of mature adipocytes promotes a strong PM GLUT4 translocation, as potent as the classic effect observed after 20-min insulin stimulation. The treatment with both E2 and insulin did not promote any further increase. The E2-induced GLUT4 translocation was confirmed via GLUT4 quantification (Western blotting) in a plasma membrane protein fraction, as well as via the increase in cellular glucose uptake [75]. Considering that E2-induced AKT activation is dependent on ESR1 translocation to the PM (analyzed under the same experimental condition, Section 7.3.1), it seems evident that E2-induced GLUT4 translocation to the PM is triggered by ESR1 shuttling to the PM [75]. Recently, the participation of ESR1 in E2-induced GLUT4 translocation was reinforced in *Esr1*-silenced adipocytes, in which the PM GLUT4 translocation was completely abolished [67]. A model of E2-induced and ESR1-mediated GLUT4 translocation to the PM is summarized in Figure 4.

## 8. Phytoestrogens

Phytoestrogens are non-steroidal natural compounds produced by plants. There are several classes of phytoestrogens spanning innumerous compounds (for a review, see [109,110]). These compounds are chemically and structurally similar to E2, providing them with the capacity to bind in both ESR1 and ESR2 [111], although their affinity for ESR1 or ESR2 can be highly variable.

Table 1 shows the most common phytoestrogens, as well as the main food sources and the relative binding activity (RBA) to ESR1 or ESR2, as compared to E2. In general, phytoestrogens display higher RBA for ESR2 than for ESR1, some of them showing strong capacity to bind in ESR1, as with genistein and coumestrol. After binding to ESR1 or ESR2, phytoestrogens can activate or block estrogen receptor ligand-binding domains, thus displaying estrogenic or antiestrogenic effects, respectively [110,111]. It is important to observe that some compounds are found in large amounts in some food sources, of which we ingest only small amounts (for example, nuts, seeds and spices); thus, their relative participation in our regular diets may be low (for a review, see [109]).

The final true role of phytoestrogens in any estrogen biological effect is still a real challenge because at least the following must be considered: (1) the ESR1/ESR2 RBA, (2) the pattern of ESR1 and ESR2 expression in the target tissue, (3) the concentration of the compound in the food source and the respective amount of intake of this food and (4) the concomitant concentration of endogenous estrogens at the target tissue. Item 4 plays an important role in women, in whom endogenous estrogen concentrations may vary from high levels (such as during pregnancy) to low levels (such as during postmenopause). It is important to highlight that the concomitant concentration of estrogen may shift the phytoestrogen effect from estrogenic to antiestrogenic.

Importantly, concerning metabolism regulation and glycemic homeostasis, the effects of some phytoestrogens might occur independently of the participation of the ESRs. For instance: (1) phloretin is a classic inhibitor of GLUT4 that has been used to block glucose transport in vitro [50]; (2) quercetin, in *ob*/*ob* T2D mice, increases *Slc2a4*/GLUT4 expression in muscle and improves glycemic homeostasis by decreasing the inflammatory response [112]; (3) resveratrol, in obese mice with T2D, also increases the *Slc2a4*/GLUT4 expression in muscle and improves glycemic control [113]. The resveratrol effect on *Slc2a4* gene expression involves epigenetic regulation of the gene: it increases the tri-methylation at lysine 9 of histone 3 (H3K9me3) in the *Slc2a4* promoter segment −498/−298, impairing the binding activity of several *Slc2a4* enhancer transcription factors [113].

Recently, the effect of diet supplementation with flaxseed or soy nuts (in the same amount) was compared with E2 replacement in ovariectomized rats. E2, but not flaxseed and soy nuts, reduced body weight; however, all treatments increased the GLUT4 content in adipose tissue, flaxseed being more effective than soy nuts [114]. These data suggest that, like E2, such diets are triggering a preponderant ESR1-mediated enhancer effect of *Slc2a4*/GUT4 expression on the adipocytes. However, the reason why only E2 altered body weight is difficult to explain, but may be related to the distinct effect on the clonal phase of adipogenesis.

In addition to phytoestrogens, we should also consider another group of ESR1/2 ligands, selective estrogen receptor modulators (SERMs), which include a large group of environmental contaminants with a potential role to stimulate or inhibit estrogenic activity. In a recent review, putative involvement of endocrine disruptor bisphenol A in Alzheimer’s disease was proposed to involve impaired GLUT4 translocation in hippocampal neurons, although there is no evidence to support this suggestion [115].

In summary, it seems that phytoestrogens also modulate *Slc2a4*/GLUT4 expression, which might alter glycemic homeostasis. As far as phytoestrogens intake is concerned, considering that they exert more powerful ESR2-mediated effects, we should expect a strong repression of *Slc2a4*/GLUT4 expression, especially in low estrogen concentration states as in postmenopausal women. In this condition, as observed in *Esr1*^−/−^ mice in which ESR2-mediated effects are preponderant [65], we should expect impaired glycemic homeostasis leading to a diabetogenic state. Conversely, if some compounds reveal preferential ESR1-mediated activity, a beneficial effect on glycemic homeostasis must be expected.

## 9. Concluding Remarks

Since long ago, clinical disorders and experimental models involving altered circulating estrogen levels have been associated to impaired glycemic homeostasis, not only in females, but also in males. However, both hyper- and hypoestrogenism could be associated to insulin resistance and DM, making the relationship between these variables hard to demonstrate. Furthermore, alterations in other hormonal systems which accompany changes in estrogen levels also delayed the demonstration of the effects of estrogen on glycemic homeostasis. The characterization of the estrogen nuclear receptors ESR1 and ESR2 finally offered a great opportunity to shed some light on the estrogen regulation of glycemic homeostasis. *Esr1*^−/−^ and *Esr2*^−/−^ mice, as well as *Cyp19a1*^−/−^ mice treated with selective ESR1 and ESR2 agonists, revealed that ESR1 activity improves glycemic homeostasis, whereas ESR2 activity impairs glycemic homeostasis, and that is accompanied by an increase and a decrease of muscle GLUT4 content, respectively. Considering that the insulin-sensitive glucose transporter GLUT4 (*Slc2a4* gene) is fundamental to insulin-regulated plasma glucose clearance, the regulations of muscle GLUT4 expression explain the regulations of glycemic homeostasis in *Esr1*^−/−^ and *Esr2*^−/−^ mice. In addition, in isolated adipocytes, it was confirmed that ESR1 enhances, whereas ESR2 represses, *Slc2a4* gene transcription. Although *Slc2a4* transcriptional regulation by isolated ESR1/ESR2 has not been demonstrated yet, their positive cooperation with SP1 and CEBPA transcription factors and their negative cooperation with NFKB transcription factor are clear. Surprisingly, in an ESR1-mediated way, E2 can induce GLUT4 translocation to the plasma membrane, increasing glucose uptake, a classic effect of insulin, fundamental to glycemic regulation. In tissues that characteristically express *Slc2a4*/GLUT4 and participate in insulin-regulated plasma glucose clearance, we must consider that ESR1 is preponderant in adipocytes whereas ESR2 is preponderant in myocytes; thus, E2 effects are opposite in muscle and adipose tissues. Considering that muscle is the major territory of plasma glucose clearance, it is expected that an increase in ESR2 activity will contribute to glycemic homeostasis impairment; moreover, a decrease in ESR1 activity, failing to counterbalance the ESR2 action, will also be deleterious to glycemic homeostasis. Additionally, the current trend for phytoestrogens intake, regarding glycemic homeostasis, is a cause for concern. In general, phytoestrogens bind preferentially in ESR2, thus showing a worrisome potential to deteriorate glycemic homeostasis.

To date, it is clear that ESR1-mediated effects are beneficial, whereas ESR2-mediated effects are detrimental to glycemic homeostasis; thus, imbalance of the ESR1/ESR2 ratio may have important consequences in metabolism. Furthermore, future considerations of clinical use of xenoestrogens and phytoestrogens must be proposed considering both their selectivity for ESR1 and ESR2 and endogenous circulating estrogen levels, invariably bearing in mind that high ESR2 activity can be dangerous for glycemic homeostasis.

## Figures and Tables

**Table 1:** Phytoestrogens: classes, main food sources, compounds and ESR1/ESR2 relative binding activity (RBA).

Classes	Main Food Sources	Compounds	RBA *	
			ESR1	ESR2
		17β-estradiol (E2)	100	100
Coumestans	Mung Beans, Soy Sprouts, Alfalfa Sprouts, Clover	Coumestrol	20	140
Isoflavones	Soy (milk, cheese, protein, tofu), Peanut, Clover, Sunflower, Seeds, Walnut	Genistein	4	87
		Daidzein	0.1	0.5
		Biochanin A	<0.01	<0.01
Flavones	Parsley, Celery, Capsicum, Citrus Peels, Pepper	Apigenin	0.3	6
		Chrysin	<0.01	<0.01
Flavanols	Beans, Tea, Spinach, Broccoli	Kaempferol	0.1	3
Chalcones	Apple, Tea, Soy-based Foods	Phloretin	0.2	0.7
Stilbenes	Grape, Wine	Resveratrol	ND	ND
Lignans	Soybean, Peanut, Broccoli, Kiwi, Banana, Cashew Nut, Orange, Flaxseeds, Cereals, Onion, Garlic	** Secoisolariciresinol	ND	
		** Matairesional	ND	

* RBA was analyzed as a ratio of concentrations of E2 and the compounds necessary to shift the binding of the specific radioligand by 50% and considering the value for E2 as 100. ** When secoisolariciresinol and matairesional, present in foods, reach the gut, they are converted into enterodiol and enterolactone, respectively, which are the final ligands of ESR1/ESR2. ND, not determined. Data are based on [109,110].
